# Editorial: Biomaterials and antibacterial materials for osseous-ligament system

**DOI:** 10.3389/fbioe.2023.1230500

**Published:** 2023-06-07

**Authors:** Dicky Pranantyo, Alideertu Dong, Liqun Xu, Yansong Qi

**Affiliations:** ^1^ Antimicrobial Resistance Interdisciplinary Research Group, Singapore-MIT Alliance for Research and Technology, Singapore, Singapore; ^2^ College of Chemistry and Chemical Engineering, Inner Mongolia University, Hohhot, China; ^3^ Chongqing Key Laboratory for Advanced Materials and Technologies of Clean Energies, School of Materials and Energy, Southwest University, Chongqing, China; ^4^ Orthopedic Center (Sports Medicine Center), Inner Mongolia People’s Hospital, Hohhot, China

**Keywords:** biomaterials, synthetic materials, natural biomaterials, antimicrobial materials, osseous-ligament system, cartilage repair, biomechanics

Trauma and infection-caused osseous-ligament defects have become a constant threat to limb function and quality of life, which inflict serious health problems to millions of people worldwide and cause major economic burdens to society (Niu et al., 2022). Severe osseous-ligament defects caused by trauma occur frequently, such as comminuted fractures, torn ligaments, and injuries with open wounds. As a burgeoning branch of tissue engineering, biomaterials for osseous-ligament regeneration face a constant risk of infection despite significant advances made over the decades in cartilage, ligament, and bone repair (Shen et al., 2022). Protection against infection as well as quality and function pose a major challenge to the science of biomaterials for tissue repair. Effective biomaterials should be of suitable quality to enhance tissue growth and restore function and should not have negative hindrances on immunity and the ability to fight infection.

In this Research Topic, we introduce the recent developments of biomaterials and corresponding therapies for the osseous-ligament systems, including synthetic materials, natural biomaterials, and antimicrobial materials. We also compile the mechanism and function of biomaterials during treatment or *in vivo* studies. On the regenerative side, we present the design and fabrication of the scaffold to better stimulate the natural spatial complexity of cell types and tissue organization of bone and joints. In terms of safety and efficacy, we discuss biomaterials integration, biocompatibility and inflammation in the host, and the successful survival of the implant in the host body.

One of the key requisites for successful implants integration and tissue engineering is maintaining the infection-free condition. Various strategies have been explored to endow antibacterial functions in osseous-ligament systems, including surface modification and smart delivery of small molecules, peptides and polymers. Kurishima et al. demonstrated that TiNbSn alloy, which exhibits low Young’s modulus, could be imparted with antibacterial properties by anodization with sodium tartrate electrolyte. Hao et al. reviewed the diversity, effects, and applications of antimicrobial peptides for bone tissue engineering. Wang et al. presented the recent progress of stimuli-responsive antibacterial materials for bone infection, with special attention to the advanced mechanisms of action to treat multidrug-resistant bacteria and biofilms. However, not all antibacterial materials are safe enough for biomedical applications. Li et al. presented a systematic review of clinical studies discussing the potential side effects of antibacterial coatings in orthopaedic implants.

This Research Topic also provides critical analyses on the established treatments for ligament and bone repairs based on the clinical data obtained from patients. Wang et al. reported a clinical study on the vertebral segments of patients to discuss whether the sizing of current cervical disc arthroplasty systems match Chinese cervical anatomic dimensions. Ren et al. studied the safety, efficacy, and functional outcomes on arthroscopic fixation of posterior cruciate ligament avulsion fracture by a bio-absorbable anchor or traditional pull-out technique. Yang et al. presented a systematic review and meta-analysis on different stem fixation methods of radial head prostheses during long-term follow-up. Cui et al. summarized the efficacy and outcome of a two-staged operation for irreducible knee dislocation with a short-term follow-up. Ma et al. reported the efficacy and medium-term outcomes of ligament advanced reinforcement system compared with auto-grafts in anterior cruciate ligament reconstruction with at least 2 years follow-up. Yang et al. conducted a histological analysis and prospective clinical trial of primary repair for treating acute proximal anterior cruciate ligament tears.

Clinical evaluations on the material and morphological designs of the applied orthopaedic and osseous-ligament implants serve as the basis of future designs and direction in tissue engineering for cartilage and bone repairs. Li et al. discussed the biomechanical performance of three Ti6Al4V volar plates with the latest designs in patients with distal radius fractures using a finite element model. Wang et al. reviewed the preparation, modification, and clinical applications of porous tantalum scaffolds, discussing the emerging manufacturing technologies, surface modification techniques, and patient-oriented designs that influenced the microstructural characteristic, bioactive performance, and clinical indications of porous tantalum implants over the past two decades. Ding et al. proposed a method for designing tuberosity reconstruction baseplate for shoulder hemiarthroplasty based on morphological data, which potentially extends the applications of biomaterial carbon fibre reinforced polymer composites in orthopaedics field.

Various supplementary elements can support osseointegration and alleviate complications, which subsequently facilitate the success of orthopaedic and osseous-ligament implants. He et al. reviewed the applications of inferior vena cava filters in orthopaedics, the current status of filters, and the progress of research into biodegradable vena cava filters, coupled with possible future developments. Zheng et al. presented a systematic review to summarize the synthetic materials and specific techniques of suture tape augmentation in anterior cruciate ligament reconstruction and evaluate the clinical outcomes. Li et al. summarized the application of additive manufacturing technology in pelvic surgery, addressing the challenges in traditional pelvic surgery, such as the complex structure of the pelvis, difficulty in exposing the operative area, and poor visibility of the surgery. Guo et al. discussed the prospects of multi-layer intelligent technologies, including smart materials, variable structures, and intelligent therapeutic planning, to achieve optimal recovery of musculoskeletal injuries.

Following osseointegration, bone repair and regeneration can be propelled by modulating stem cell differentiation and chondrocyte redifferentiation. Kong et al. demonstrated that titanium dioxide nanotubes layers promote osteogenic differentiation through nuclear localization of Yap and downstream activation of Piezo1 expression. Yang et al. presented a bibliometric and visualization analysis of stem cell therapy to facilitate meniscus regeneration in the past decade. Li et al. incorporated osteogenic mesenchymal stem cells (MSC) into gelatin microcryogels to form microtissues and applied these self-assembled microtissues to induce osteogenesis for *in vivo* bone regeneration.

From the compilation of these individual contributions, the collective articles are broad in nature, ranging from initial research to clinical applications that address the challenges of antibacterial strategy and tissue engineering in bone repair. This Research Topic therefore provides references for ongoing research in natural and synthetic biomaterials (hydrogels, fibers, surfaces, coatings, polymers, peptides, and peptidomimetics) for osseous-ligament reconstruction. It encourages future directions in antibacterial functions, modulation of stem cell differentiation and chondrocyte redifferentiation, promotion of survival and integration of implants in ligaments and bone, additive manufacturing of scaffolds for tissue engineering of bone and cartilage, and translational research on tissue engineering of bone and joints.

## References

[B1] Niu X. Li N. Du Z. Li X. (2022). Integrated gradient tissue-engineered osteochondral scaffolds: Challenges, current efforts and future perspectives. Bioact. Mater 20, 574–597. 10.1016/j.bioactmat.2022.06.011 35846846 PMC9254262

[B2] Shen Q. Qi Y. Kong Y. Bao H. Wang Y. Dong A. (2022). Advances in copper-based biomaterials with antibacterial and osteogenic properties for bone tissue engineering. Front. Bioeng. Biotechnol. 9, 795425. 10.3389/fbioe.2021.795425 35127670 PMC8811349

